# Neurocytomes centraux: corrélations cliniques et radiopathologiques à propos de 12 observations

**DOI:** 10.11604/pamj.2017.27.222.12016

**Published:** 2017-07-25

**Authors:** Fayçal Abbad, Souad Sellami, Fe Hazmiri, Najat El Idriss Ganouni, Said Ait Benali, Mouna Khouchani, Hanane Rais

**Affiliations:** 1 Service d’Anatomie et Cytologie Pathologiques, Hôpital Ar Razi, CHU Mohammed VI, Marrakech, Maroc; 2 Service de Radiologie, Hôpital Ar Razi, CHU Mohammed VI, Marrakech, Maroc; 3 Service de Neurochirurgie, Hôpital Ar Razi, CHU Mohammed VI, Marrakech, Maroc; 4 Service de Radiothérapie, Centre d’Onco-Radiothérapie et Hématologie, CHU Mohammed VI, Marrakech, Maroc

**Keywords:** Neurocytome central, radiologie, histopathologie, Central neurocytoma, radiology, histopathology

## Abstract

Les neurocytomes centraux sont définis comme étant des néoplasmes composés de cellules rondes, uniformes, ayant un profil immunophénotypique neuronal et un index de prolifération bas. Elles représentent 0,5% des tumeurs intracrâniennes. Elles sont habituellement situées à proximité du foramen de Monro et peuvent être à l’origine d’une hydrocéphalie obstructive. Nous rapportons 12 observations de neurocytomes centraux colligés au laboratoire d’anatomie pathologique du CHU Mohammed VI de Marrakech entre janvier 2006 et juin 2015. Le but de ce travail est de rapporter et décrire les aspects radio-pathologiques de ce type histologique rare. Le sex-ratio homme/femme était de 1,4. L’âge moyen au diagnostic était de 22,3 ans. La symptomatologie révélatrice était dominée par l’hypertension intracrânienne chez tous nos patients, associée à une baisse de l’acuité visuelle et une diplopie. Notre matériel d’étude a concerné une biopsie simple dans un cas, une exérèse subtotale dans sept cas et une exérèse totale dans quatre cas. L’étude histopathologique a montré une prolifération tumorale d’architecture endocrine. Les cellules tumorales sont uniformes de petite taille le plus souvent. L’index mitotique était bas. Cette prolifération tumorale s’accompagnait d’un fond fibrillaire et d’un réseau vasculaire développé de type arborescent. L’étude immunohistochimique était identique chez tout les patients. Elle a montré une positivité des cellules tumorales à l’anticorps anti synaptophysine, à chromogranine et NSE. Chez tous nos patients la corrélation radiopathologique était en faveur d’un neurocytome central (Grade II – OMS 2016). A travers cette série, nous rapportons les particularités anatomo-cliniques, radiologiques et évolutives de ces tumeurs rares.

## Abstract (en)

Central neurocytomas are defined as neoplasms composed of round, uniform cells, with neural immunophenotypic profile and low proliferation index. They account for 0.5% of intracranial tumors. They usually occur near the foramen of Monro and can cause obstructive hydrocephaly. We conducted a retrospective data collection from records of 12 patients with central neurocytomas in the Anatomopathological Laboratory at the Mohammed VI University Hospital, Marrakech, between January 2006 and June 2015. This study aimed to report and describe the radiopathological features of this rare histologic type. The male/female sex-ratio was 1.4. The average age at diagnosis was 22.3 years. The revealing symptomatology was dominated by intracranial hypertension associated with decreased visual acuity and diplopia in all patients. Our study concerned about simple biopsy in one case, subtotal resection in seven cases and total resection in four cases. Histopathological examination showed tumor proliferation with endocrine architecture. Tumor cells are more often small and uniform. Mitotic index was low. Tumor proliferation was associated with fibrillary fundus and vascular network with three types of trees. Immunohistochemical examination was identical in all patients. It showed tumor cells positive for anti-synaptophysin antibodies, chromogranine and NSE. In all patients the radio-pathologic correlation suggested central neurocytoma (grade II - WHO 2016). This study highlights the anatomo-clinical, radiological and evolutionary features of these rare tumors.

## Introduction

Les neurocytomes centraux sont classées parmi les tumeurs neuronales et neuro-gliales mixtes. Elles sont définies par l’OMS 2016 comme étant un néoplasme intraventriculaire rare. Elles sont composées de cellules rondes, uniformes, avec un profil immunophénotypique neuronal et un index de prolifération bas [1]. Ces tumeurs sont habituellement situées à proximité du foramen de Monro. Elles ont été décrites initialement par Hassoun et al. en 1982 [1]. Il s’agit d’une tumeur cérébrale rare. Elle représente seulement entre 0,25–0,5% des tumeurs intracrâniennes. Les neurocytomes centraux réalisent le plus souvent des tableaux clinicoradiopathologique indispensable pour le diagnostic. Elles surviennent chez le sujet jeune et ont siège supratentoriel (ventricule latéral et ou troisième ventricule). La ressemblance histopathologique avec l’oligodendrogliome ou l’épendymome et le profil immunohistochimique neuronal sont des élements fondamentaux qui permettent d’identifier cette entité [2]. A travers notre série, nous rapportons le profil de ces tumeurs diagnostiquées dans notre service.

## Méthodes

Nous avons réalisé une étude rétrospective transversale sur une période de onze ans depuis janvier 2006 à juin 2015 portant sur une série de douze patients porteurs d’un neurocytome central. Ce diagnostic a été confirmé histologiquement au service d’anatomie pathologique du CHU Mohammed VI. Les données complémentaires ont été recueillies à partir des dossiers des patients aux services de radiologie, de neurochirurgie et d´oncologie radiothérapie du CHU Mohammed VI de Marrakech, Maroc.

## Résultats

Notre série comporte douze patients, âgés entre 17 et 28 ans avec un âge moyen de 22,3ans, dont 7 femmes et 5 hommes. Un tableau d’hypertension intracrânienne était présent chez tous les malades. Des troubles visuels (baisse de l’acuité visuelle, diplopie binoculaire, œdème papillaire) ont été trouvés dans 72% des cas. D’autres signes ont été retrouvés (Ataxie, hémiparésie droite, Hypotonie fébrile). L’approche chirurgicale initiale a consisté en une biopsie simple dans un cas ; en une exérèse subtotale dans sept cas et en une exérèse totale chez quatre patients (Tableau 1).

**Tableau 1:** Caractéristiques cliniques et thérapeutiques de notre série

Numéro de patient	Age (ans)	Sexe	Symptomatologie clinique initiale	Resection chirurgicaleTotale/ subtotale
**1**	24	F	HTIC baisse de l’acuité visuelle œdème papillaire	Biopsie simple Tumeur de grande taille inaccessible
**2**	21	H	HTIC, Ataxie, Hémiparésie droite, baisse de l’acuité visuelle	Subtotale
**3**	28	F	HTIC, Hypotonie febrile	Subtotale
**4**	21	H	HTIC	Subtotale
**5**	18	F	HTIC, diplopie binoculaire	totale
**6**	22	F	HTIC, baisse de l’acuité visuelle	Subtotale
**7**	25	H	HTIC	Subtotale
**8**	18	H	HTIC	Totale
**9**	23	F	HTIC	Subtotale
**10**	27	F	HTIC	Subtotale
**11**	17	H	HTIC	Totale
**12**	24	F	HTIC	Totale

**Figure 1:**
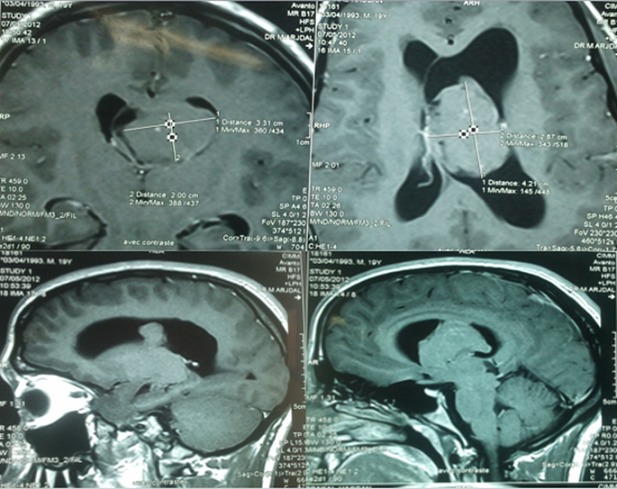
IRM en séquence T1 après gadolinium en montrant un neurocytome central du corps du ventricule latéral

La tomodensitométrie a mis en évidence des calcifications dans trois cas, un rehaussement modéré et hétérogène chez six patients. L’hydrocéphalie a été retrouvée dans six cas. L’imagerie par résonance magnétique a permis de visualiser l’accolement de la tumeur au septum pellucidum dans quatre cas, au ventricule latéral dans quatre cas et au plancher dans deux cas. Le signal tumoral était hyperintense T2, hétérogène en «bulle de savon» dans sept cas (Figure 1). Un foyer hémorragique intratumoral a été retrouvé dans trois cas. Le rehaussement tumoral était hétérogène chez tous nos patients. Les caractéristiques TDM et IRM des neurocytomes explorés sont résumées dans les Tableau 2 et Tableau 3. La corrélation radiopathologique était de mise dans tous les cas. Elle a montré une concordance totale entre l’évocation diagnostique et la confirmation pathologique.

**Tableau 2:** caractéristiques tomodensitométriques de notre série

	Densité		Calcifications	PDC	Hydrocéphalie
**Aspect en imagerie**	isodense	hyperdense	+	+	+
**Nombre**	4	6	3	10	6

**Tableau 3:** caractéristiques à l’imagerie par résonance magnétique de la série

Signal				Topographie		Insertion			Extension		
Hétérogene/ bulle de savon	Kystique	hémoragie	PDC	VLD	VLG	septum	Latéral	plancher	V3	V4	Extra-ventricuaire
7	4	3	10	6	4	4	4	2	3	1	1

**Figure 2:**
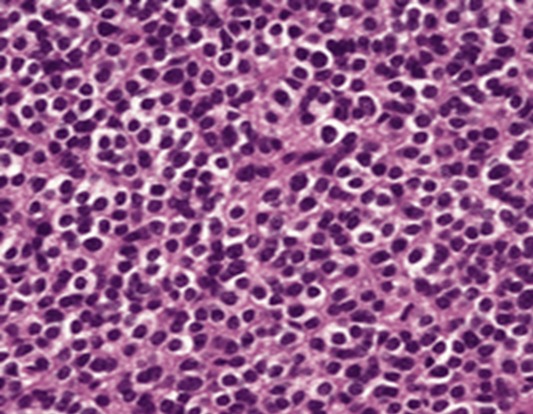
Prolifération tumorale d’architecture endocrinoïde faites de cellules arrondies au cytoplasme clair (Hématéine Eosine x20)

L’examen histopathologique, chez tous nos patients a retrouvé un schéma architectural commun avec des variations propres à chaque cas. Il s’agissait de nappes de cellules monomorphes de petite taille (Figure 2). Dans cinq cas, elles étaient de type oligodendroglial (munies d’un noyau central arrondi large et d’un cytoplasme clair). La densité cellulaire était modérée dans dix cas et élevée dans les deux autres cas. Le stroma était fibrillaire plus ou moins abondant avec une vascularisation arborescente développée dans tous les cas. L’activité mitotique est faible et la nécrose tumorale était absente dans tous les cas. Les calcifications on été retrouvées dans 16% des cas. A noter que des variations de densité cellulaires et architecturales ont été retrouvées ou sein d’une même prolifération tumorale. Dans 75% des cas, le diagnostic a été évoqué sur la morphologie seule. Un diagnostic différentiel a été suggéré dans trois cas, avec un épendymome dans deux cas et avec une tumeur glioneuronale papillaire dans l’autre cas.

**Figure 3:**
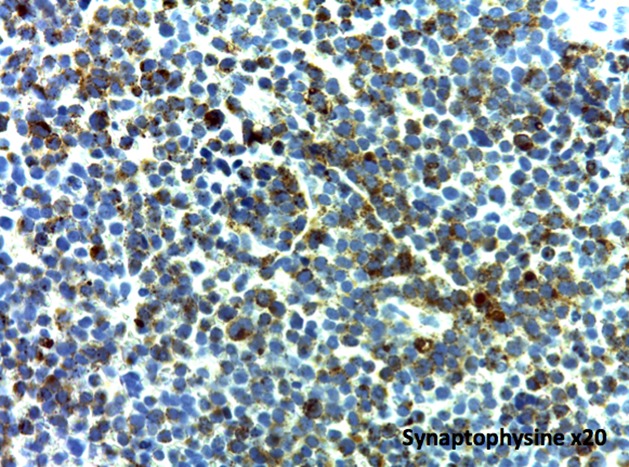
Expression cytoplasmique intense et diffuse des cellules tumorales à l’anticorps anti-Synaptophysine (Hématéine Eosine x20)

**Figure 4:**
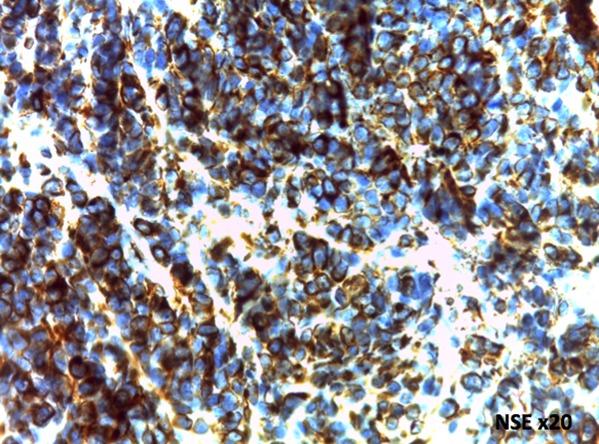
Expression cytoplasmique intense et diffuse des cellules tumorales à l’anticorps anti-Neuron Specific Enolase (Hématéine Eosine x20)

L’étude immunohistochimique était réalisée chez tous nos patients. L’expression de la synaptophysine est exprimée de façon diffuse dans onze cas (Figure 3), la NSE (Neuron specific enolase) dans quatrecas (Figure 4), de la chromogranine dans deux cas et de la GFAP dans quatres cas (astrocytes incorporés dans la tumeur). L’index de prolifération Ki-67 n’a pas excédé 1% dans toute notre série.

Les suites opératoires immédiates étaient simples dans 75% des cas. Deux patients ont présenté des saignements per opératoire, compliqués de décès et un cas d’hémorragie intra ventriculaire compliqué d’hémiparésie droite. Six patients ont reçu une radiothérapie. Trois patients ont été perdus de vue. La survie à cinq ans était de 83% des cas dans notre série.Aucune récidive n’a été rapportée.

## Discussion

Le neurocytome central a été démembré par Hassoun et al. en 1982 [1]. Il décrit alors une tumeur neuronale, survenant chez l’adulte jeune au niveau du troisième ventricule. Cette tumeur possède des caractéristiques pathologiques distinctes des neuroblastomes cérébraux et pouvant mimer un oligodendrogliome sur le plan morphologique. Ces tumeurs étaient initialement décrites comme des épendymomes du foramen de Monro. L’histogenèse de ces tumeurs reste inconnue. Au vu de l’implication fréquente du septum pellucidum et d’une différenciation neuronale, le neurocytome central pourrait provenir des cellules précurseur de la matrice germinale périventriculaire. Celles ci ont aussi un potentielde faible prolifération.

C’est une tumeur rare, correspondant au grade II de l’OMS 2016 [2,3]. Elle représente 0,25 % des tumeurs cérébrales. Cette incidence est probablement sous-estimée vu sa confusion avec d’autres tumeurs ventriculaires en particulier avec l’oligodendrogliome. L’âge moyen de diagnostic est de diagnostic est de 28,5 ans avec des extrêmes allant de 1 an à 80 ans [2,4]. Les cas pédiatriques rapportés sont exceptionnels [5].Les deux sexes sont touchés de façon équivalente. Notre série est caractérisée par un âge de survenue plus jeune et une prédominance féminine.

La symptomatologie révélatrice est faite d’hypertension intracrânienne avec possibilité de troubles cognitifs. L’histoire de la maladie est courte généralement moins de trois mois [6,7]. D’autres signes peuvent être observés : une baisse de l’acuité visuelle avec un œdème papillaire, des troubles mnésiques, des troubles de la marche, un déficit neurologique ou des crises comitiales. Ces deux derniers signes témoignent en général d’un potentiel agressif. L’aspect en imagerie du neurocytome central est évocateur [8,9]. L’aspect habituel est celui d’une tumeur souvent lobulée, bien délimitée, hétérogène et prenant le contraste. Des calcifications peuvent être retrouvées de façon inconstante. La tumeur siège dans le ventricule latéral, dans la région du foramen de Monro, dans près de 70 % des cas. Cette proportion est retrouvée dans notre série. L’atteinte ventriculaire bilatérale est notée dans 13 % des cas. L’extension au troisième ventricule est décrite dans 25 % des cas. La localisation exclusive au niveau du troisième ventricule a été décrite dans seulement 3 % des cas. L’extension vers le quatrième ventricule est exceptionnelle [9].

La tomodensitométrie cérébrale objective une tumeur de localisation intraventriculaire aux contours nets. Celle ci est d’emblée isodense ou légèrement hyperdense. Elle se modifie modérément après injection de gadolinium. Les calcifications sont observées dans 50% des cas et sont souvent amorphes ou globuleuses. L’IRM délimite mieux la tumeur et précise son insertion, généralement il s’agit d’une tumeur isointense en séquence pondérée en T1. Le signal est variable en T2, mais souvent isointense ou hyperintense par rapport au cortex cérébral. Les zones d’hyposignal T1 et T2 présentes au sein de la lésion correspondent à des calcifications ou à de l’hémorragie. Les zones d’intensité hétérogène correspondant aux microkystes, à des vaisseaux dilatés et de calcifications [7,9].

Le diagnostic différentiel se pose avec toutes les tumeurs intraventriculaires, telles que l’épendymome comme deux cas dans notre série, l’oligodendrogliome, le papillome du plexus choroïde, l’astrocytome. Macroscopiquement, le neurocytome central est une tumeur blanchâtre, lobulée, bien circonscrite. C’est une tumeur friable montrant fréquemment des remaniements nécrotiques, calcique et kystiques.

Sur le plan histopathologique, le neurocytome se caractérise par la présence de cellules uniformes, petites et moyennes à noyaux arrondis ou réguliers. La chromatine est finement granulée « en poivre et sel». Les nucléoles sont discrets. Dans certaines zones, de petites cellules avec des halos périnucléaires sont retrouvées. Elles peuvent évoquer l’oligodendrogliome [10,11]. Dans la majorité des cas rapportés, l’activité mitotique est réduite voire nulle et la nécrose est inexistante. La vascularisation sous jacente est faite de petits vaisseaux à paroi fine de disposition arborescente. Tous ces aspects ont été retrouvés dans notre série.

D’autres aspects architecturaux sont décrits plus rarement : Les cellules peuvent être étroitement agencées mais peuvent également avoir un fond finement « neuropile-like ». Par endroits, les tumeurs présentent des zones cellulaires denses alternant avec des zones fibrillaires/acellulaire. Les derniers éléments sont principalement périvasculaires et ont une matrice de neuropile fibrillaire donnant un aspect de pseudo-rosettes simulant un épendymome [12]. La présence de fortes similitudes à l’histologie et en imagerie avec l’oligodendrogliome et l’épendymome imposeconstamment le recours à l’immunohistochimie. L’étude immunohistochimique est nécessaire pour un diagnostic précis. Le neurocytome central exprime le Neuron Specific Enolase, la synaptophysine, la protéine S100, la leu-7 et la calcineurine. Les neurocytome centraux sont GFAP, Neurofilament, vimentine, INI-1 et p53 négatif. Quelques cellules évoquant plutôt des astrocytes incorporés dans la lésion peuvent exprimer la GFAP [12,13]. L’indice de prolifération Ki67 est faible, inférieur à 1 %. Ce qui est retrouvé dans notre série. D’exceptionnelles formes malignes peuvent présenter les critères habituels de malignité et un indice de prolifération plus élevé [13].

Il n’existe aucun marqueur spécifique des neurocytomes centraux. Le diagnostic de certitude repose sur un faisceau d’arguments cliniques, radiologiques, histopathologiques et immunohistochimiques. Sur le plan moléculaire, ces tumeurs comportent de nombreuses altérations génomiques. La surexpression du gène N-Myc et des gènes impliqués dans la voie de signalisation WNT ont été mis en évidence. Aucune codélétion 1p/19q n’a été rapportée dans ces tumeurs [2,13]. La résection chirurgicale la plus complète possible est le traitement de choix. Elle doit être réalisée sans entraîner une grande morbidité [14]. Un suivi neuroradiologique à moyen et long terme régulier s’avère nécessaire en postopératoire, soit pour la recherche d’une récidive si l’exérèse est complète soit pour surveiller l’évolution d’un résidu si l’exérèse est partielle [15,16].

La radiothérapie est souvent proposée comme traitement adjuvant en cas d’exérèse incomplète. La dose délivrée en cas de neurocytome central typique est de 50 à 54 gray en 30 fractions pendant six semaines. La radiochirurgie par gamma-knife offre un grand avantage par rapport à la radiothérapie conventionnelle vu que la tumeur est bien limitée et présente une cible idéale pour la radiochirurgie [17]. Des auteurs ont rapporté un cas de réduction marquée du volume tumoral et un cas de régression totale, le diagnostic a été établi par biopsie stéréotaxique. Le pronostic très favorable. La durée de survie moyenne sans récidive est de 79,3 mois. Les récidives tumorales surviennent chez 10 % des patients en moyenne à 18 mois de la chirurgie [18]. Elles ont été corrélées à un index de prolifération Ki-67 de plus de 2 %, témoignant de l’agressivité de la tumeur. Dès que l’index de prolifération dépasse les 3 %, il existe un retentissement significatif sur la progression et l’évolutivité locale mais aussi sur la survie du patient [19]. Des cas rarissimes de transformation maligne ont été rapportés avec un Ki67 à plus de 40%.

## Conclusion

Les neurocytomes centraux sont des tumeurs rares d'origine neuronale, de grade II de l’OMS 2016. Elles sont caractérisées par une croissance lente et une localisation intraventriculaires. La démarche diagnostique repose sur une approche pluridisciplinaire. La corrélation clinicoradiopathologique, l’apport de la morphologie et du profil phénotypique permettent d’écarter les diagnostics différentiels. L'exérèse chirurgicale totale est associée à unbon pronostic. La radiothérapie postopératoire est considérée dans les cas d'exérèse incomplète. Des formes exceptionnelles agressives ont été rapportées. Elles sont associées constamment à un index de prolifération Ki67 élevé. Le pronostic est alors sombre.

### Etat des connaissances actuelles sur le sujet

- Les neurocytomes centraux sont des tumeurs rares du système nerveux central considérées comme bénignes;
- Plusieurs formes morphologiques existent et l’approche diagnostique est basée sur la corrélation radiopathologique;
- Le traitement est essentiellement chirurgical, la radiothérapie complémentaire peut être prescrite.

### Contribution de notre étude à la connaissance

- Il s’agit d’une série descriptive originale;
- Cette série souligne et affirme l’intérêt d’une corrélation radiopathologique;
- Nous rapportons les aspects diagnostiques, thérapeutiques et évolutifs chez nos patients et les confrontons aux données de la littérature.

## Conflits d’intérêts

Les auteurs ne déclarent aucun conflit d’intérêts.
